# First record of *Anopheles stephensi* in Sri Lanka: a potential challenge for prevention of malaria reintroduction

**DOI:** 10.1186/s12936-017-1977-7

**Published:** 2017-08-10

**Authors:** A. G. Gayan Dharmasiri, A. Yashan Perera, Jeevanie Harishchandra, Hemantha Herath, Kandasamy Aravindan, H. T. R. Jayasooriya, Gaya R. Ranawaka, Mihirini Hewavitharane

**Affiliations:** 1 Anti Malaria Campaign Headquarters, Public Health Complex, Narahenpita, Colombo 5, Sri Lanka; 2 Regional Malaria Office, Anti Malaria Campaign, Mannar, Sri Lanka; 3 Department of Zoology, The Open University of Sri Lanka, Nawala, Nugegoda Sri Lanka

**Keywords:** *Anopheles stephensi*, Malaria, Prevention of malaria re-introduction, Sri Lanka

## Abstract

**Background:**

The major malaria vector in Sri Lanka is reported to be *Anopheles culicifacies* with *Anopheles subpictus, Anopheles annularis*, and *Anopheles varuna* considered as potential vectors. The occurrence of *Anopheles stephensi*, which is the key vector of urban malaria in India and the Middle East, had never been reported from Sri Lanka.

**Methods:**

A series of entomological investigations were carried out by the Anti Malaria Campaign, Ministry of Health, Sri Lanka during December 2016 to April 2017 in two localities of the Mannar District in the Northern Province of the country. Adult mosquito collections were done through indoor and outdoor resting collections, animal and human biting collections and emergence traps. Potential mosquito breeding sites were investigated through larval surveys. The larvae and adults of *An. stephensi* were initially identified using morphological keys, and subsequently confirmed by sequencing the barcode region of the cytochrome c oxidase I (*COI*) gene.

**Results:**

This is the first report of the presence of *An. stephensi* in the island of Mannar in the Northern Province of Sri Lanka. *Anopheles stephensi* (36.65%) was the most abundant anopheline species in the larval habitats in Mannar. It was found breeding together with *An. culicifacies* (20.7%), *An. subpictus* (13.5%) and *An. varuna* (28.13%). *Anopheles stephensi* was found to be abundantly breeding in built wells used for domestic purposes. Adult females of *An. stephensi* were observed in emergence trap collections (93.9%), human landing catches all night (79.2%), pyrethrum spray sheet collections (38.6%), outdoor collections (8.3%), donkey-baited trap collections (14.3), and cattle-baited net trap collections (0.7%).

**Conclusions:**

Sri Lanka was certified as malaria-free by the WHO in September 2016, however, this new finding may pose a serious challenge to the efforts of the Ministry of Health to prevent the re-introduction of malaria transmission in the country, considering the role that *An. stephensi* could play in urban and high vulnerability areas of Sri Lanka.

## Background

Sri Lanka, an island in the Indian Ocean, achieved zero transmission of locally acquired malaria cases in November 2012. Following completion of three consecutive malaria-free years, Sri Lanka received the certification of elimination of malaria from the World Health Organization in September 2016. In the past few decades, malaria was a major public health problem, being endemic in 22 districts of the intermediate and dry zones of the country [1]. In Sri Lanka, the major vector of malaria is reported to be *Anopheles culicifacies*, and *Anopheles subpictus, Anopheles annularis* and *Anopheles varuna* are considered as potential vectors [2, 3].

With the cessation of local malaria transmission in the country, disease surveillance along with entomological surveillance have been strengthened to prevent the re-introduction of malaria. The largest threat for re-introduction of malaria into the country is the importation of malaria cases from endemic countries [4]. The influx of malaria parasites into the country are through security personnel returning from United Nations peace missions, asylum seekers coming through United Nations High Commissioner for Refugees (UNHCR), foreign workforces in different trades, and local migrants going to malarious countries on travel, business or pilgrimage. The abundance of local malaria vectors in the formerly malarious areas, even in the absence of malaria transmission, has made the country both vulnerable and receptive to the re-introduction of malaria [5].

Sri Lanka has a strong entomological surveillance system, since the 1930s, which has evolved based on the changing epidemiology of malaria transmission in the country. Apart from the period 2008–2014, during which a private organization (Tropical Environmental Diseases and Health Associates Pvt. Ltd (TEDHA)) assisted in several of the conflict-affected districts in Northern Province, including Mannar, the entomological activities on malaria vectors are conducted by the Anti Malaria Campaign (AMC), Sri Lanka.

Since achieving malaria-free status, the AMC has continued its efforts on parasite and vector surveillance and on activities to find any new threats. As such, malaria surveillance programmes are routinely conducted by the AMC in the previously malarious areas and entomology teams have been especially vigilant on the possibilities of finding invasive malaria vectors in the country. During one of these surveillance studies in December 2016, evidence for the presence of *Anopheles stephensi* in Sri Lanka was first reported from two sites in the Mannar Island. Mannar District comprises both mainland Mannar and Mannar Island, and the latter is connected to the mainland of Sri Lanka through a causeway. A 50-km chain of limestone shoals between Talaimannar, located on Mannar Island and Rameshwaram Island of India, separates Sri Lanka from the Indian subcontinent. This limestone shoal is referred to as the Adam’s Bridge, which has historical importance and provides geographical evidence of the former land connection between India and Sri Lanka.

*Anopheles stephensi*, belonging to the subgenus *Cellia* and Neocellia series, had never been reported from Sri Lanka until the current study first revealed evidence for its occurrence in two localities in Mannar District in December 2016. The latest available taxonomic key for the identification of *Anopheles* fauna of Sri Lanka published in 2016 described 23 *Anopheles* species [6]. This most recent identification key, nor those previously published by Carter [7], Amerasinghe [8], Amerasinghe [9], or Gunathilaka [10] included *An. stephensi* as a species found in Sri Lanka. However, *An. stephensi* is found in Afghanistan, Bangladesh, China, India, Indochina, Iran, Iraq, Myanmar, Pakistan, Taiwan, and Thailand [11]. This species is considered an important vector of *Plasmodium falciparum* and *Plasmodium vivax* throughout its natural range and is the main urban malaria vector of India, Pakistan, Iran, and Iraq [12–14].

Considering the importance of *An. stephensi* as an urban vector of falciparum and vivax malaria in other countries, its breeding on Mannar Island could pose a serious threat to preventing the re-introduction of malaria and its transmission in Sri Lanka. Therefore, in December 2016, entomological surveillance in Mannar District was intensified for four consecutive months with special focus on six selected sampling blocks on Mannar Island. Larval and adult surveys were conducted with the primary aim of confirming the presence and bionomics of *An. stephensi* in Sri Lanka.

## Methods

### Sampling sites

Mannar District is situated in Northern Province of Sri Lanka (08° 52′N and 80° 04′E) and has an extent of 1996 sq km. Its population is 99,051 (2012 Census) with a population density of 50/km^2^. The district is one of the areas that was heavily affected by the 30-year conflict in the country and Sri Lankan refugees returning from India are resettled here.

The entomological field investigations were carried out from December 2016 to April 2017 on Mannar Island as a part of risk group-based entomological surveillance. Two localities, Murugan Kovil and Pesalai, which are 1 km apart, belonging to Pesalai Public Health Inspector area of Mannar Medical Officer of Health, were initially surveyed. This site was named Block C and was selected as a sentinel site and sampled during the 5 months for larval and adult *An. stephensi* at monthly intervals using the sample techniques described below. The other sites were located throughout the Mannar Island, in six blocks, namely Taleimannar, Nadukuda, Wankalipadu, Olaithuduwal, Erukkalampiddi, and Eluttur. These sites were named as Block A, B, D, E, F and G, respectively. The sampling locations and blocks are shown in Fig. 2. In selecting these sites, factors such as resettled population returning from India, population aggregation, availability of breeding sites, and feasibility of carrying out field mosquito collections were considered.

### Larval sampling

Larval surveys (LS) were carried out using standard dipping (volume of the dip 350 ml) method in the potential mosquito breeding habitats in all Blocks A to F. Dipping was done following WHO guidelines and standard operating procedures for entomological surveillance techniques of AMC, Sri Lanka [15, 16]. The anopheline larvae collected were recorded according to their stage of larval development as either first to second instars (early), third to fourth (late) instar. The immature density of each habitat was calculated as density of larvae per 100 dips, i.e., total number of larvae/number of dips taken × 100.

### Adult mosquito sampling

Indoor-resting adult mosquitoes were sampled by spray sheet collections (PSC) using pyrethrum to knock-down, and by indoor hand collections (IHC) using mouth aspirators for indoor-resting mosquitoes. Animal-baited trap collections were done by cattle-baited trap collections (CBTC) and donkey-baited trap net collections (DBTC). An experimental hut was constructed in Block A using coconut leaves to thatch the walls and roof to collect adult mosquitoes. Cattle and donkeys were used as baits inside the hut. In both net trap and hut trap collections an animal was introduced after sunset and mosquitoes trapped inside were collected before sunrise. Emergence trap collections (ETC) were carried out by fitting a window trap onto the mouth of a well and covering the remaining area with polythene. Human landing catches (HLC) were carried out at 18.00–06.00 and at 18.00–21.00. These sampling techniques followed the standard operating procedures for entomological surveillance techniques of AMC, Sri Lanka [15]. An overview of sampling techniques deployed in each sampling site is shown in Table 1.

**Table 1:** Overview of sampling blocks, monitoring period and work output of each field technique

Block	Localities sampled for anophelines	Monitoring time period	Larval surveys	Adult surveys								
			Number of dips	1	2	3	4	5	6	7	8	9
A	Talaimannar	Jan 2017–Apr 2017	1527	−	2	−	−	13.75	10	−	18	−
B	Nadukuda	Jan 2017–Apr 2017	1160	−	5	−	−	−	15	−	12	−
C	Pesalai	Dec 2016–May 2017	5565	11	15	2	10	28.66	76	29.19	108	282
D	Wankalaipadu	Jan 2017–Apr 2017	0	4	14	1	−	3.75	22	6	60	72
E	Erukkalampiddy	Jan 2017–Apr 2017	3840	−	−	−	−	−	−	−	−	−
F	Olaithuduwal	Jan 2017–Apr 2017	2056	0	3	−	−	−	15	−	18	−
G	Elluthur	Jan 2017–Apr 2017	2658	13	5	−	−	5	−	0.66	12	−

1: ETC; 2: CBTC; 3: DBTC; 4: CBHC; 5: IHC in man hours; 6: Number of rooms tested for PSC; 7: ODC in man hours; 8: HLC (partial) in total man hours; 9: HLC (all night) in total man hours

### Morphological identification of *Anopheles* spp.

Third and fourth instar larvae collected from larval surveys were morphologically identified to species level using compound microscopes and taxonomic keys prepared by Amerasinghe [9]. In the event of failure to identify the *Anopheles* larvae using the above taxonomic key, they were kept until emergence of adults. Larvae were kept in plastic larval trays filled with water from the breeding sites from they were collected and fed with larval feed mixture of 1:1:1 tuna meal, bovine liver powder and vitamin mix. Adults emerging 1–4 days later were killed using diethyl ether. Double mounts were prepared using pinning method. The collected live adult mosquitoes from the adult sampling collections and emerged adults from larvae were identified to species level using achromatic magnifying lenses (×10), and mounted dead specimens were identified using a stereomicroscope. If there was an *Anopheles* specimen that did not match with morphological features in the key prepared by Amerasinghe, taxonomic keys for identification of Indian anophelines were used to identify both larvae and adults [8, 17–20].

### Genetic identification of *Anopheles stephensi*

Following photo-documentation, four adults morphologically identified as *An. stephensi* that had emerged from larval collections, were subjected to molecular study using the barcode region of the COI gene [21, 22]. One sample was collected from Block A in January 2017 (9° 2N, 79° 52E), two samples were collected from Block B in April 2017 (9° 4N, 79° 50E), and the fourth sample was collected from Block E in April 2017 (9° 4N, 79° 49E). Genomic DNA was extracted using DNeasy blood and tissue ^®^ DNA extraction kit (Qiagen, Germany) according to the procedure supplied by manufacturer. The *COI* barcode region of approximately 700 bp was PCR amplified using primers developed by Kumar et al. for mosquitoes [23]. The PCR products were purified using Wizard^®^ SV Gel and PCR Clean-up System (Promega, USA) and were sequenced at a commercial sequencing facility (Macrogen, Korea). The sequences were analysed for sequence identity using BLAST analysis [24] and by using ‘Identification Request’ function in BOLD [25].

## Results

### First record of *Anopheles stephensi* in Sri Lanka

Initial larval surveys carried out in Block A in December 2016 enumerated four *Anopheles* species: *An. culicifacies, An. subpictus*, *An. varuna* and an unidentified species (Table 2). The larvae that could not be identified initially and the adults emerging from them were later morphologically identified as *An. stephensi.*

**Table 2:** Composition of *Anopheles* species collected by entomological sampling techniques in Block A study site in December 2016

*Anopheles* species	Number (percentage) collected by each technique		
	Larval survey	Indoor hand collections	Cattle-baited net trap collections
*An. culicifacies*	77 (41.2%)	1 (100%)	–
*An. stephensi*	105 (56.2%)	–	–
*An. subpictus*	–	–	11 (100%)
*An. varuna*	5 (2.7%)	–	–
Total	187	1	11

*Anopheles stephensi* larvae were morphologically identified based on the following characters: presence of a long prothoracic pleural hairs and both being simple; one mesothoracic pleural hair being simple and the other branched; both metathoracic pleural hairs being branched; strongly developed palmate hairs on abdominal segments *III*–*VII* with leaflets and various filaments; thoracic palmate hair being very weakly palmate; palmate hair of abdominal segment *I* being very weakly palmate, having between 3 or 5 branches. This last feature helped to specifically differentiate these *An. stephensi* larvae from those closely resembling *Anopheles maculatus* larvae, as the latter possesses more than five branches.

**Fig. 1:**
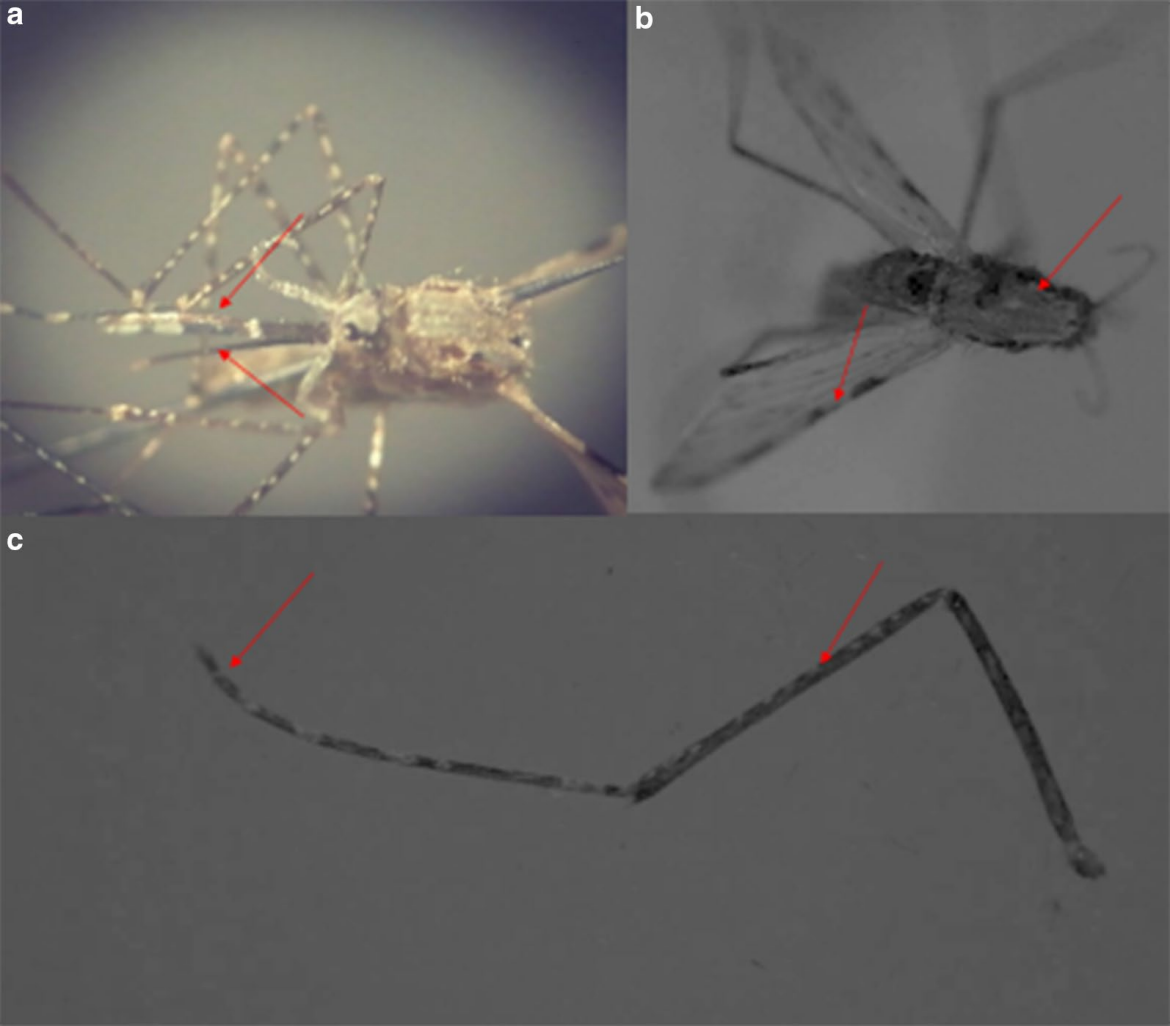
Morphology of *Anopheles stephensi* adult female collected from Mannar Island. **a** Banding pattern and speckling of palpi and appearance of proboscis. **b** Thorax showing broad bands of white scales and wing. **c** Hind leg showing speckling of leg and fifth tarsomere having only a narrow white band

The adult females were identified as *An. stephensi* based on the following morphological characters (Fig. 1): adult females were medium in size with a wing length of 2.5 mm; similar to all *Anopheles s*pecies, adult females of *An. stephensi* had maxillary palps as long as their proboscis; proboscis was dark in colour and pale only at the tip; the costa on the wing had four dark spots that extended to vein 1 of the wing; maxillary palps had three pale bands; one pale band was apical in position on the maxillary palp and the other pale band was sub-apical in position; third apical band was more proximal in position on the maxillary palpi compared to the apical and the sub-apical bands; the apical and sub-apical pale bands were equal in size and were separated by an intermediate dark band; speckling of palps was visible in between sub-apical pale band and the most proximal pale band on the maxillary palps; thorax was covered by pale scales and broad bands were prominent (Fig. 1b); all legs were speckled; hind tarsomeres of the adults had short bands of pale scales and hind tarsomere 5 was dark (Fig. 1c). These characteristics helped to exclude any possible misidentification with the morphologically closely related *An. maculatus*.

### Genetic confirmation

*COI* sequences of the four specimens analysed (683 bp) were confirmed as *An. stephensi* for sequence identity by BLAST and BOLD analysis. These sequences have been deposited in GenBank under the accession numbers MF124608-MF124611. All four samples showed 99–100% homology to previously submitted sequences (DQ154166.1 and AF425844.1) of *An. stephensi COI* gene in BOLD and GenBank.

### Findings of entomological surveys

Analysis of data collected through the entomological survey in all six sampling blocks from December 2016 to May 2017 revealed the presence of 11 *Anopheles* species from Mannar Island. There were 4213 larvae and 3018 adult *Anopheles*: *Anopheles barbirostris, An. culicifacies, Anopheles jamesii, Anopheles nigerrimus, Anopheles peditaeniatus, Anopheles pallidus, Anopheles tessellatus, An. stephensi, An. subpictus, An. varuna*, and *Anopheles vagus*.

Eight *Anopheles* were encountered in the larval surveys (Table 3) and *An. stephensi* was the most abundant followed by *An. varuna* and *An. culicifacies. Anopheles stephensi* was breeding in built wells and barrels used for water storage purposes.

**Table 3:** *Anopheles* species caught in different larval habitats

*Anopheles species*	Number of anophelines caught in different breeding sites (%)					
	Wells	Cement tanks	Barrels	Ground pools	Lagoon margin	Total
*An. barbirostris*	10 (0.3)	0	0	0	0	10 (0.24)
*An. culicifacies*	797 (21.6)	41 (100)	0	34 (7.5)	0	872 (20.7)
*An. jamesii*	0	0	0	22 (4.9)	0	22 (0.52)
*An. pallidus*	3 (0.1)	0	0	0	0	3 (0.07)
*An. stephensi*	1506 (40.9)	0	38 (100)	0	0	1544 (36.65)
*An. subpictus*	179 (4.9)	0	0	392 (86.9)	0	571 (13.55)
*An. varuna*	1182 (32.1)	0	0	3 (0.7)	0	1185 (28.13)
*An. vagus*	6 (0.2)	0	0	0	0	6 (0.14)

**Fig. 2:**
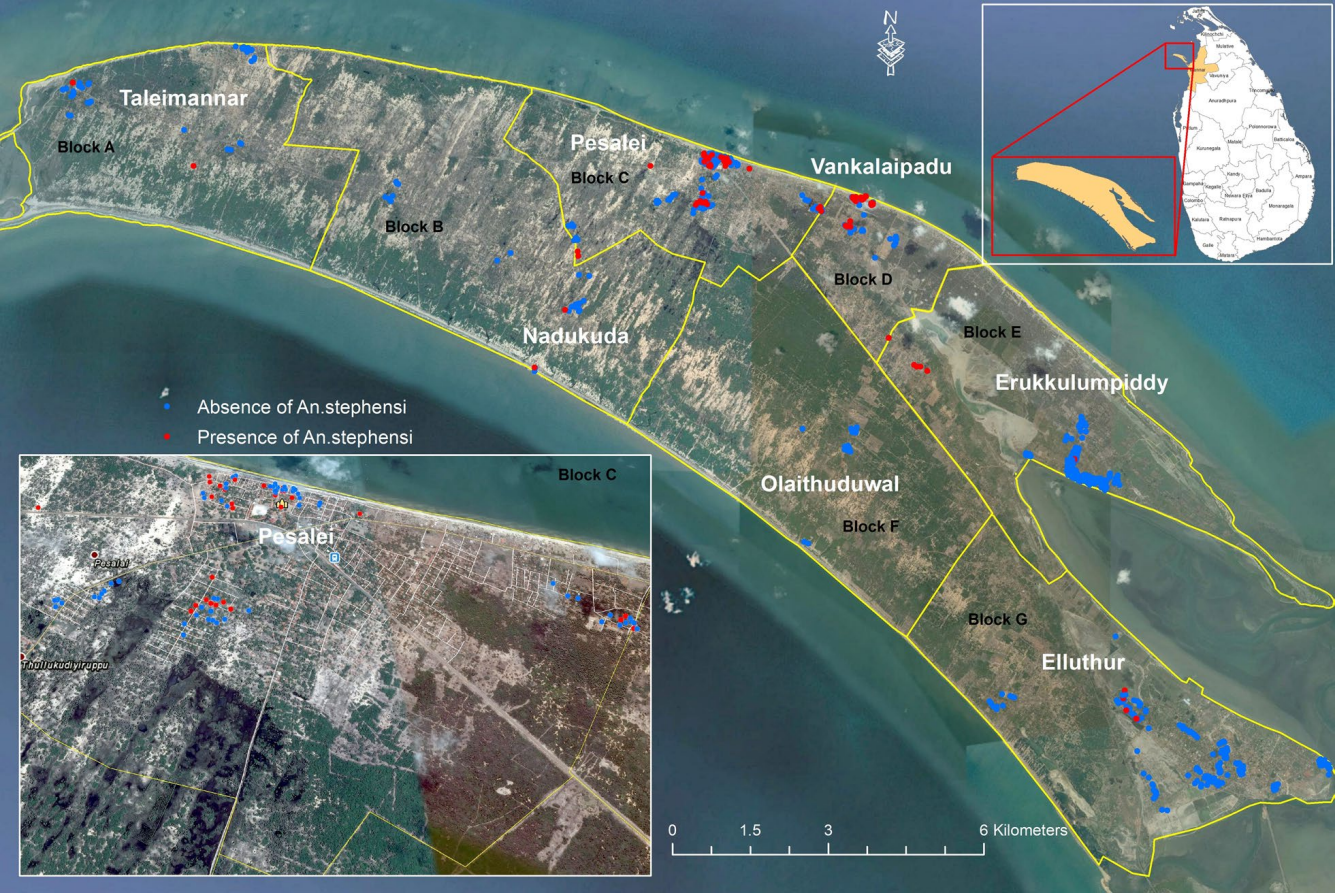
Map of *Anopheles stephensi* breeding wells on Mannar Island

Because wells were found to be the most productive and abundant breeding site for *An. stephensi*, the preference of malaria vectors to breed in such habitats was further investigated. These wells were not very deep and most were built with cemented inner walls. A total of 892 wells were surveyed in the study period and 472 were found to be breeding sites for malaria vectors (Table 4). Distribution of the wells are mapped in Fig. 2.

**Table 4:** Details of wells where malaria vectors breed

Malaria vector	Number (%) of wells found with malaria vector larvae		
	Alone	In combination with other *Anopheles*	Total
*An. stephensi*	112 (12.55)	33 (3.69)	145 (16.25)
*An. culicifacies*	69 (7.73)	70 (7.84)	139 (15.58)
*An. varuna*	104 (11.65)	56 (6.27)	160 (17.93)
*An. subpictus*	19 (2.13)	9 (1.01)	28 (3.13)
Total	304 (64.4)	168 (35.6)	472 (100)

*Anopheles stephensi* was found in seven out of the nine techniques deployed for study of the bionomics of this species (Table 5). Indoor- and outdoor-resting and zoophilic and anthropophillic behaviours were observed during the 6-month period. *Anopheles subpictus* was the most abundant species found among the 11 *Anopheles* species encountered in different adult mosquito collection techniques.

**Table 5:** Numbers of *Anopheles* species caught in different adult sampling techniques

*Anopheles* species	Number collected by each technique (%)									
	1	2	3	4	5	6	7	8	9	Total
*Anopheles barbirostris*	−	−	−	−	2 (0.1)	−	−	−	−	2 (0.1)
*Anopheles culicifacies*	5 (6.1)	3 (23.1)	1 (100)	4 (33.3)	29 (1.0)	2 (16.6)	−	1 (9.1)	1 (4.2)	46 (1.5)
*Anopheles jamesii*	−	−	−	−	59 (2.1)	−	−	−	−	59 (1.9)
*Anopheles nigerrimus*	−	−	−	−	57 (2.0)	−	−	−	−	57 (1.9)
*Anopheles peditaeniatus*	−	−	−	−	25 (0.9)	−	1 (2.4)	−	−	25 (0.8)
*Anopheles pallidus*	−	−	−	−	15 (0.5)	−	−	−	−	15 (0.5)
*Anopheles stephensi*	77 (93.9)	5 (38.5)	−	1 (8.3)	20 (0.7)	−	6 (14.3)	3 (27.8)	19 (79.2)	131(4.3)
*Anopheles subpictus*	−	5 (38.5)	−	6 (50)	2542 (90.1)	10 (83.3)	35 (83.3)	7 (63.6)	4 (16.7)	2609 (86.4)
*Anopheles tessellatus*	−	−	−	1 (8.3)	15 (0.5)	−	−	−	−	16 (0.5)
*Anopheles vagus*	−	−	−	−	16 (0.6)	−	−	−	−	16 (0.5)
*Anopheles varuna*	−	−	−	−	41 (1.45)	−	−	−	−	41 (1.35)
Total	82	13	1	12	2821	12	42	11	24	3018

1: ETC; 2: PSC; 3: IHC; 4: ODC; 5: CBTC; 6: CBHC; 7: DBTC; 8: HLC—partial night; 9: HLC—full night

## Discussion

*Anopheles stephensi* is an important urban malaria vector in neighbouring countries, such as India and Pakistan, but has not before been reported from Sri Lanka. This is the first report of its presence in Sri Lanka based on the entomological surveillance studies that detected larvae and adults of the species Mannar Island, located in the dry zone area of the country. Presence of *An. stephensi* was confirmed on morphological identification and molecular evidence based on the DNA barcoding approach using the *COI* gene.

There is limited information on malaria vector surveillance studies carried out in the Northern Province of Sri Lanka prior to 2010, including in Mannar District, because of the 30-year war in that area. This lack of information is a major constraint in determining in absolute terms the absence of this particular species in the area. The most recent vector surveillance study in Mannar District was during 2010–2012, where Gunathilaka et al. studied *Anopheles* species composition and breeding habitat diversity, however, the presence of *An. stephensi* in Mannar District was not revealed in that study [26]. The Sri Lankan mosquito checklist has not been updated, following the attempts made by Jayasekara and Chelliah [27] and the dichotomous key prepared by Amerasinghe, until more recent attempts in 2014 and 2016; none of these studies reported *An. stephensi* in the country [6, 8–10]. Because of this gap in knowledge it is unclear whether *An. stephensi* has been undetected on Mannar Island or whether this is a recent invasion. Invasion from a neighbouring Indian state may be a possibility since *An. stephensi* has been incriminated as the main vector of urban malaria in India, and has shown a southwards spread on the mainland due to rapid urbanization and water storage practices. *Anopheles stephensi* has caused malaria outbreaks in Kerala State, India where such epidemics were not reported until 1996, and it is now reported breeding in water storage tanks in Lakshadweep islands off the coast of Kerala, indicating possibilities of invasion of islands [11, 28, 29].

Similarly, *An. stephensi* in Mannar Island breeds in water storage containers, mainly in domestic wells with cemented walls, where the highest percentage of breeding was recorded. Further co-existence of *An. stephensi* with *An. culicifacies* and *An. varuna* was also observed in wells*. Anopheles stephensi* breeding with other mosquito vectors such as *Aedes aegypti, Aedes albopictus, Culex quinquefasciatus,* and *Culex vishnui* has also been observed in studies in India [11]. In the absence of a continuous supply of water throughout the year, villagers, new settlers returning from India, and workforces involved in construction work and fishing depend on well water which has become an ideal breeding site for *An. stephensi.* Domestic wells with cemented walls appear to be permanent water bodies with the potential for breeding even in the dry season. It was also apparent that plastic barrels used by the local population for water storage were another potential breeding site which adds complexity to the situation. This container breeding habit of *An. stephensi* predominantly in wells, overhead or ground level water tanks, cisterns, tanks, coolers, roof gutters, curing pits in construction areas, fountains and ornamental tanks in urban areas has been observed in other countries [30]. Use of other small containers may be expected during the wet season.

*Anopheles stephensi* is considered an invasive species, which enters new towns and settlements. There is evidence of *An. stephensi*-related urban malaria in the African country, Djibouti due to introduction of *An. stephensi* from Asia. Suspected routes for this introduction were through transportation of goods of refugee returnees from the nearest geographically endemic peninsula [31]. Therefore, it is possible that the presence of *An. stephensi* is an invasion into Sri Lanka from India. The distance between Dhanushkodi in India and Talaimannar (Block B) in Sri Lanka is only 50 km, through the Palk Strait. There have been various attempts by fishermen of both countries to cross the borders for fishing. In Pesalai (Block A) the Sri Lanka Navy had informed the Regional Malaria Officer to carry out vector surveillance and vector control in the fishing boats of Indian fishermen who had in three instances crossed the border. Intersectoral collaboration with security personnel for vector surveillance activities in Mannar is an example of a recent initiative taken by the Ministry of Health in its efforts to keep Sri Lanka malaria free. Water storage utensils of the fishing boats may be potential breeding grounds for *An. stephensi* and the inside wall surfaces of these boats may harbour adult mosquitoes.

Although entomological surveys conducted on Mannar Island revealed the presence of *An. stephensi*, no evidence has been found for its presence in the mainland at present. Further investigations of the distribution of this species especially in the mainland are required, including in urban areas, and the AMC must put more emphasis on rigorous entomological surveillance in adjoining districts in a rapid and progressive manner to restrict further expansion of distribution.

*Anopheles stephensi* control on Mannar Island by antilarval measures was initiated using 1% Temephos granules 1 mg/l and larvivorous fish *Poecilia reticulata* [32], and the resettled population in this area were provided with long-lasting insecticidal nets, Yorkool^®^. In planning appropriate chemical-based vector control, knowledge of bionomics and biology of a vector species is an essential requirement. It must be a national priority to prevent further establishment of *An. stephensi* on Mannar Island and to curtail its spread to other parts of the country. A rigorous vector control strategy should be implemented with the support of other sectors to increase the awareness of local people of the potential threat of malaria re-introduction by this dangerous vector species. Closing abandoned wells, covering wells to prevent mosquito egg laying, and introduction of larvivorous fish can be carried out by the local population if a behavioural change awareness campaign were to be introduced. In addition to the health sector, the support of local authorities, the environmental sector and non-governmental organizations is of paramount importance in practising control interventions.

## Conclusions

This paper describes the first confirmed report of *An. stephensi* in Sri Lanka. The post-war resettlement taking place in Northern Province, coupled with invasion of malaria vectors with or without parasites, may cause a serious threat to sustaining malaria-free status in Sri Lanka. Because *An. stephensi* is known to be a vector of urban malaria, if this species spreads to urban areas of Sri Lanka, it will have important implications for transmission of malaria as urban malaria has not previously been observed in Sri Lanka.

Urban malaria is quite a different situation to rural malaria in terms of transmission of the disease and disease risk assessments due to diversity of ecologies, exposure patterns and prevention opportunities, etc. [29]. Increasing numbers of people becoming residents of urban environments may cause prevention and control of malaria activities to be location specific. To minimize this risk of malaria transmission by *An. stephensi* in what appears to be an urban malaria situation on Mannar Island, not only should its further spread be controlled through vector control measures, its distribution should be monitored throughout Sri Lanka through active entomological surveillance. In mitigation of the present situation, the Ministry of Health needs to initiate a solid action plan to prevent the re-introduction of malaria into the country considering the potential of spread and establishment of *An. stephensi* into other areas of the country, and particularly considering the possible role it could play in transmission of urban malaria.

## Abbreviations

AMC: Anti Malaria Campaign
*COI*: cytochrome oxidase subunit 1
UNHCR: United Nations High Commissioner for Refugees
TEDHA: Tropical Environmental Diseases and Health Associates Pvt. Ltd
ETC: Emergence Trap Collections
PSC: Pyrethrum Spray sheet Collections
IHC: Indoor Hand Collections
ODC: Outdoor Collections
CBTC: Cattle-Baited Trap net Collections
CBHC: Cattle-Baited Hut trap Collections
DBTC: Donkey-Baited Trap net Collections
HLC: Human Landing Catches
LS: Larval Surveys
NCBI: National Center for Biotechnology Information
